# Investigating the Timing and Extent of Juvenile and Fetal Bone Diagenesis in a Temperate Environment

**DOI:** 10.3390/biology12030403

**Published:** 2023-03-03

**Authors:** Amanda R. Hale, Ann H. Ross

**Affiliations:** 1 SNA International for Defense POW/MIA Accounting Agency, Joint Base Pearl Harbor-Hickam, Oahu, HI 96853, USA; 2 Department of Biological Sciences, North Carolina State University, Raleigh, NC 27695, USA

**Keywords:** bone diagenesis, juvenile skeletal remains, histotaphonomy

## Abstract

**Simple Summary:**

Understanding bone diagenesis, or alteration, in juvenile and fetal remains has important implications for forensic science. First, it can suggest information about the deposition of the remains and a possible postmortem interval (PMI). Second, it can assist in evaluating bone integrity and the potential for molecular testing of these remains for forensic purposes. This study investigates how early bone diagenesis is observed in fetal and juvenile mammalian remains as well as differences in degradation based on the deposition of the remains (e.g., blanket wrapping, shallow burial, etc.). We found that there were differences in the extent of bone diagenesis between depositions, with bagged remains exhibiting relatively less degradation over time than the other three depositions, while buried remains exhibited the greatest extent of degradation over time. However, all the remains showed bone diagenesis regardless of time of interment or deposition, with all remains exhibiting alteration as early as three months. This is consistent with adult remains, although the presentation of alteration differs and is likely related to developmental differences between subadult and adult bone.

**Abstract:**

It is well understood that intrinsic factors of bone contribute to bone diagenesis, including bone porosity, crystallinity, and the ratio of organic to mineral components. However, histological analyses have largely been limited to adult bones, although with some exceptions. Considering that many of these properties are different between juvenile and adult bone, the purpose of this study is to investigate if these differences may result in increased degradation observed histologically in fetal and juvenile bone. Thirty-two fetal (n = 16) and juvenile (n = 16) *Sus scrofa domesticus* femora subject to different depositions over a period of two years were sectioned for histological observation. Degradation was scored using an adapted tunneling index. Results showed degradation related to microbial activity in both fetal and juvenile remains across depositions as early as three months. Buried juvenile remains consistently showed the greatest degradation over time, while the blanket fetal remains showed more minimal degradation. This is likely related to the buried remains’ greater contact with surrounding soil and groundwater during deposition. Further, most of the degradation was seen in the subendosteal region, followed by the subperiosteal region, which may suggest the initial microbial attack is from endogenous sources.

## 1. Introduction

Macroscopic observations during decomposition are the result of underlying cellular processes [1,2,3,4,5,6,7]. Historically, forensic science research has focused on the factors that correlate with macroscopic observations for the purpose of estimating the postmortem interval [5,6,8,9,10,11]. However, with advancing biomolecular analyses, more recent studies have turned to investigating the causes of decomposition at the microscopic and molecular levels for two primary reasons [2]. First, it aids in identifying samples that have been exposed to greater diagenetic alteration that, in turn, could have degraded or altered molecules of interest (e.g., DNA preservation) [2,12,13,14,15]. Second, studies have found significant variation in the environmental factors that drive gross observations of decomposition across climates and even sites [6,16,17,18,19,20]. Histological observations, however, have presented consistent morphologies of microscopic destruction across archaeological sites and environments, often regardless of time scale and differences in soil microbiota [14,21,22,23,24,25,26,27]. Thus, the factors that contribute to the microscopic destruction of bone may be less variable, providing more useful information regarding taphonomic history as well as identifying more ideal samples for molecular analysis.

Bone diagenesis is influenced by both intrinsic and extrinsic factors mediated by biological, physical, and chemical processes [28,29,30]. Autolytic microbial activity primarily mediates soft tissue decomposition [5,7,31]. However, chemical processes must act in concert to allow for the microbial invasion of bone. The spatial organization of fully mineralized collagen provides resistance to microbial attack, and the spatial organization is dependent upon the bone’s intrinsic composition [25,32,33,34]. Bone is comprised of an organic component, a mineral component, and water [35]. The mineral component is bone apatite, which contributes to the structure and strength of living bone through its chemical association with collagen [28,32,35]. This spatial relationship between collagen and bone apatite contributes to the microporosity of fresh bone (approximately < 8 nm), which prevents the access of collagenases, thus inhibiting bacterial and fungal enzymes [25,36,37]. However, when chemical processes alter the mineral component (e.g., ion exchange), the organic component can typically then be accessed by microbes [33,38,39].

Chemical degradation of the mineral component is thought to primarily occur via interactions with the surrounding soil and groundwater that promote the dissolution of bone apatite, which increases bone porosity [25,40,41]. Chemical alterations that have been noted are changes in crystallinity and ion exchange with exogenous ions found in the surrounding soil and groundwater [25,28,42]. All these processes can result in diagenetic alteration of the mineral component, which then allows microbial and fungal enzymes access to the organic component [25,31,43,44].

These diagenetic alterations have primarily been studied in adult bones from archaeological contexts [23,24,26,45,46,47]. It is well understood that the intrinsic factors that contribute to bone diagenesis are those that also influence bone density during life, including sex, age, and disease; these affect the relative proportions of the organic and mineral components [21,28,48,49,50,51]. Therefore, for immature bones, special consideration must be paid to the relationship between the organic and mineral components during development. Immature bone is composed primarily of the organic component with a relatively reduced mineral component [48]. As this influences bone porosity, these intrinsic properties can reduce bone survivability and likely contribute to the lesser representation of juvenile remains in bioarcheological contexts, likely mediated by microbial attack [21,28,50,51,52,53]. Of note, Caruso and colleagues [52] identified evidence of microbial attack in juvenile archeological remains that was primarily driven by their intrinsic properties and only secondarily by their burial environment.

Microbial attack is one of the earliest biological processes to affect bone preservation [24,25,31,44,54]. Bell and colleagues [14] found evidence of microbial invasion in adult bones from forensic contexts as early as three months postmortem. White and Booth [22] found evidence of microbial attack as early as six months in their sample of neonatal and juvenile pig remains. The histological observation of microbial attack was first described by Wedl [55] but expanded and defined by Hackett [44] as Wedl, or centrifugal tunneling, resulting from fungal attack and non-Wedl microscopical focal destruction (MFD), ascribed to bacterial attack. There are three morphological types of MFD described by Hackett [44]: ‘linear longitudinal’, ‘budded’, and ‘lamellate’. These morphologies can be distinguished histologically due to their size, shape, the presence of a hyper-mineralized rim, and the presence of lamellate content [44]. Jans and colleagues [24] found that MFD was observed more often in complete burials, and fungal alteration was often observed in fragmentary remains, such as butchered animals. They hypothesize that the presence of MFD in complete burials most likely occurred during early decomposition and putrefaction [24]. This is supported by White and Booth [22], who found that the early occurrence of microbial alteration in their juvenile and neonatal samples is most likely the result of endogenous microbial attack during the early postmortem period.

Regardless of the endogenous or exogenous origin of the invading bacteria, these results suggest that juvenile and fetal remains may be generally more susceptible to microbial attack. However, the previous studies on juvenile and neonatal (or fetal) remains have only investigated those exposed on the surface or interred in shallow burials. It is currently unknown how various depositions can influence the timing and extent of microbial attack in juvenile and fetal remains. The study presented here investigates variation in the timing and extent of tunneling as evidence of microbial attack in juvenile and fetal *Sus scrofa domesticus* remains from four depositions with seasonal exposure times between three months and two years.

## 2. Materials and Methods

### 2.1. Depositional Environment

Thirty-two (16 juvenile and 16 fetal) *Sus scrofa domesticus* remains were selected for histological analysis from a larger study [16] as a proxy for human remains due to compositional similarities [56]. The remains were obtained from the North Carolina State University swine farm immediately prior to each seasonal deposition. *Sus scrofa domesticus*, on average, has a body mass greater than 5 kg; they are a readily available analog, and they provide a general mammalian model for bone anatomy and histology. Juvenile pigs having a mass of 15.9 and 22.7 kg were used as proxies for human children up to 9 years of age, and fetal pigs having a mass of 1.8 and 2.7 kg were used as proxies for human neonatal remains. The remains were interred seasonally, beginning in June 2013 and ending in the spring of 2015, after a total study period of 755 days (about 2 years). The traditional calendar for the start of each season was used as the initial day of placement. One pig per deposition was placed each season: one juvenile was placed on the surface (n = 8), one was buried (n = 8), one fetal pig was placed inside a plastic garbage bag (n = 8), and one was wrapped in a cotton baby blanket (n = 8). All pigs were deposited immediately following euthanization and a bone mineral density scan. Surface remains were enclosed in cages to mitigate scavenging where possible; however, the bagged fetal remains from winter 2013 were consumed by scavengers, leaving 31 pigs for the histotaphonomic component of the study.

Deposition seasons were summer, fall, winter, and spring, with the average temperatures classifying this region as a Cfa climate according to the Köppen-Geiger climate classification for 1980–2016 [57,58]. A Cfa climate is considered temperate, without a dry season and a hot summer, which comprises 13.4% of the climatic variation in North America [58]. Weather data were obtained from the State Climate Office of North Carolina Lake Wheeler Road Field Lab weather station, located one-quarter mile from the field site. Data is freely available for download on their website. Table 1 provides the details regarding the date of deposition each season, the calculated accumulated degree days (ADD) for the surface remains, and the calculated ADD for the buried remains from the daily soil temperature, mean soil temperature, and mean soil moisture.

During deposition, the surface juvenile, bagged fetal, and blanket-wrapped fetal were photographed and scored for soft-tissue decomposition. The buried juvenile sample was not accessed until recovery. After the two-year study period, all the remains were collected for histotaphonomic analyses. This allowed for the exposure of remains in each deposition between three months and two years. For a full analysis of the soft tissue decomposition of these remains, see Ross and Hale [16].

### 2.2. Histological Methodology

After collection, a femur was sampled for histological thick sections from each of the 31 pigs in this study. The preparation of the histological samples followed published methods [59]. The samples were embedded in plastic resin to preserve them and ensure sample integrity during slide preparation. One-millimeter-thick sections were produced using a Buehler Isomet 1000 (Buehler, Lake Bluff, IL, USA) saw with a 15 high concentration (HC) diamond-edged blade. Each thick-section wafer was ground to a final thickness of 50–75 µm on a Buehler™ variable-speed grinding unit (Buehler, Lake Bluff, IL, USA) with a diamond disc. Then, each section was mounted on a glass slide with a coverslip using SECUREMOUNT mounting media (Buehler, Lake Bluff, IL, USA). The following information was recorded on each slide: (1) slide identifier, (2) element name, (3) element side, and (4) anatomical orientation. One thick section per bone was produced, resulting in 31 midshaft femoral thick sections.

All the cross-sections were examined for signs of microscopic degradation, both qualitatively and quantitatively. Initially, the observed degradation of the samples was quantitatively assessed following the Oxford Histological Index [26], and results are reported elsewhere [16]. However, after observing more extensive micro-focal destruction, the ordinal tunneling index (TI) reported by White and Booth [22] was adapted as a quantitative measure of destruction because it had previously been applied to immature *Sus scrofa* remains and showed good interobserver reliability. Table 2 provides a description of the four index scores adapted for this study. All the sections were scored, and observations regarding the primary location of observed degradation were noted (i.e., periosteal, subperiosteal, subendosteal, or endosteal regions).

### 2.3. Data Analysis

A destructive degradation model was applied to examine the relationship between ADD and TI. This statistical procedure is used to model product deterioration over time. A log-logistic distribution was selected as it is more appropriate for decomposition studies (i.e., measuring the degradation of a product over time) that exhibit logistic patterns. This model distribution examined the relationship between the response or degradation method (i.e., TI) and the time variable (ADD). The common path with intercept model was selected because it fits a single distribution whose location parameter changes linearly over time. All statistical analyses were conducted in JMP 16.0 [60]:

(1) $$\mu =b_{0}+b_{1}\times f\left(\mathrm{time}\right)$$

where µ represents the mean observations, b_0_ represents the slope of the distribution, b_1_ represents TI, and time represents the ADD measure.

The mean soil temperature and mean soil moisture were calculated from the daily soil temperature and soil moisture data for all days each of the remains were exposed to investigate if there were significant correlations between the environmental and temporal data and the bone bioerosion observed for each specimen (see Table 1). A partial least squares regression model was used to investigate the relationship between the dependent (TI) and independent variables (ADD, mean soil temperature, and mean soil moisture) for each deposition. These variables were compared because any exogenous microbes would be derived from the surrounding soil, and these variables could affect bacterial composition. Finally, a simple linear regression was performed to examine the correlation between each of the three independent variables (ADD, mean soil temperature, and mean soil moisture) and TI.

## 3. Results

**Figure 1:**
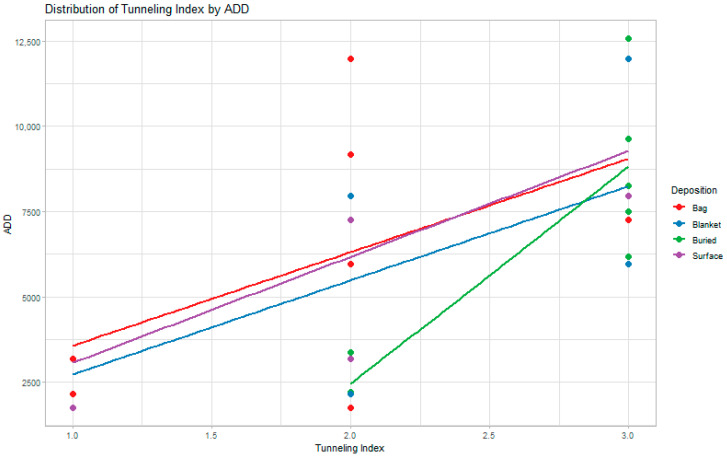
A scatter plot illustrating the distribution of TI scores over time (time represented by ADD) by deposition. Best-fit simple regression lines are here to demonstrate the relationship between bacterial bioerosion and time.

**Figure 2:**
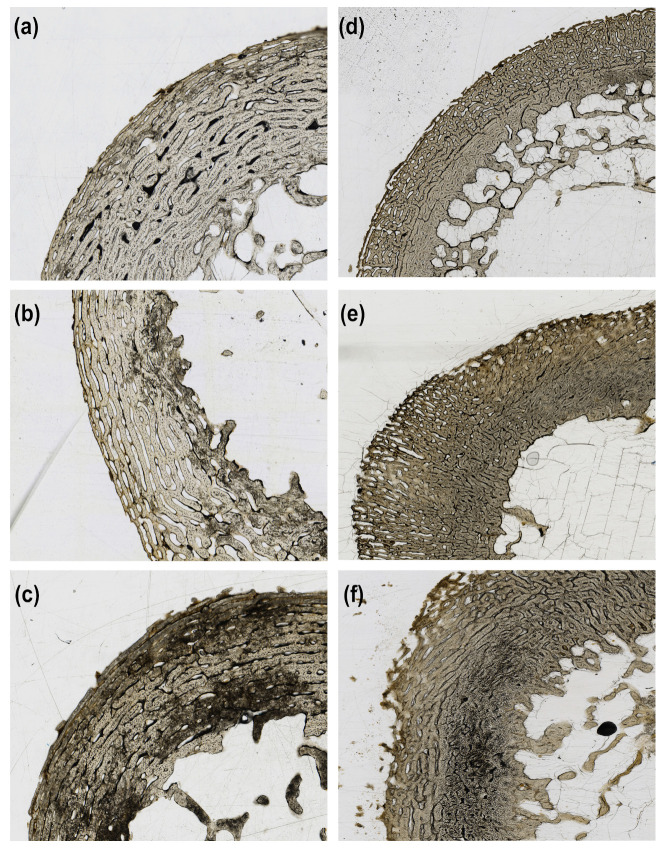
Examples of tunneling index (TI) scores 1 to 3 for both fetal and juvenile sections from this study. (**a**) Fetal sample with a TI score of 1; (**b**) Fetal sample with a TI score of 2; (**c**) Fetal sample with a TI score of 3; (**d**) Juvenile sample with a TI score of 1; (**e**) Juvenile sample with a TI score of 2; (**f**) Juvenile sample with a TI score of 3. No samples were scored as 0 with no damage.

The TI scores were variable, with the buried juveniles showing the most consistent pattern of increasing TI scores as time exposed increased, but all depositions showed an increase in TI scores over time (Figure 1). Figure 2 presents examples of TI scores 1 to 3 for both fetal and juvenile samples from this study. In samples with less microstructural damage (TI = 2), the damage was observed most often in the subendosteal region, and those with more damage (TI = 3) presented damage in either the subendosteal and subperiosteal regions or as a coalescence between these two regions, often with little to no damage along the endosteal and periosteal surfaces. The samples with lower scores showed an even distribution of enlarged osteocyte lacunae across the entire cross-section than those with more extensive damage to the microstructure, which showed a more variable pattern (i.e., when more damage was observed, it was more concentrated and less diffuse across the section than those with generally less damage present). Notably, all of the samples exhibited damage to the microstructure regardless of time exposed, and no buried samples had a TI less than 2.

**Figure 3:**
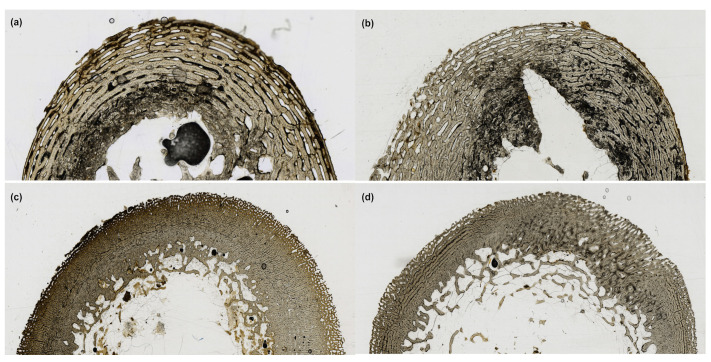
The histological sections from each deposition that were exposed for the approximate ADD from the destructive degradation probability model. All were deposited during the first spring season, with the bagged fetal, blanket fetal, and surface juvenile remains represented here having an ADD of 7246.89. The buried juvenile remains represented here have an ADD of 7494.78. (**a**) Bagged fetal cross-section (TI = 3); (**b**) Blanket fetal cross-section (TI = 3); (**c**) Buried juvenile cross-section (TI = 3); (**d**) Surface juvenile cross-section (TI = 2).

Table 3 presents the probabilities calculated by the destructive degradation model and Bayesian information criterion (BIC) of each log-logistic model. The destructive degradation model shows that there is a positive linear relationship between TI and ADD for all depositions. For the bagged fetal remains, the degradation profile shows that the predicted TI is 1.81 at an ADD of 6860.15 with a 95% prediction interval of 0.89–3.69. The crossing-time distribution profile shows that there is a 63% probability that the TI score will be 2 at 6860.15 ADD. For the blanket fetal remains, the degradation profile shows that the predicted TI is 2.27 at an ADD of 6860.15 with a 95% prediction interval of 1.25–4.16. The crossing time distribution profile shows that there is a 31% probability that the TI score will be 2 at 6860.15 ADD. For the buried juvenile remains, the degradation profile shows that the predicted TI is 2.67 at an ADD of 7153.78 with a 95% prediction interval of 2.15–3.30. The crossing time distribution profile shows that there is a 25% probability that the TI score will be 2.5 at 7153.78 ADD. For the surface juvenile remains, the degradation profile shows that the predicted TI is 2.01 at an ADD of 6860.15 with a 95% prediction interval of 1.24–3.23. The crossing time distribution profile shows that there is a 50% probability that the TI score will be 2 at 6860.15 ADD. The model performed best with the bagged fetal remains, with a 63% probability that at 6860.15 ADD, the TI would be 2 or having only minor portions of the microstructure affected by amalgamated enlarged osteocyte lacunae. Figure 3 illustrates the sections that have TI scores consistent with the predicted TI score at the approximate ADD (i.e., 6860.15 and 7153.78). None of the sections was scored as having no damage, but the fetal remains showed the least amount of microstructural damage related to bacterial tunneling. Table 4 presents the model from each deposition’s destructive degradation profile.

Table 5 presents the model estimates from the partial least squares regression model for the dependent variable, TI, and the independent variables (mean soil temperature, mean soil moisture, and ADD). The partial least squares model shows that for all depositions, the independent variables (ADD, mean soil temperature, and mean soil moisture) were not significantly associated with the TI scores. However, for the bagged fetal remains, the mean soil temperature had a *p*-value (0.06) close to the level of significance (α = 0.05) that suggests it may have had some influence on the bone bioerosion observed for this deposition. Further, the bagged fetal remains model showed the best linear fit (R^2^ = 0.83) between TI and the independent variables, followed closely by the model for the buried juvenile remains (R^2^ = 0.81). Both the blanket fetal and surface juvenile remains showed a moderately linear relationship between TI and the independent variables (R^2^ = 0.54 and 0.51, respectively).

When ADD, mean soil temperature, and mean soil moisture were considered independently in the simple linear regression models, the bagged fetal remains showed a significant correlation with mean soil temperature (0.85, *p* = 0.016). All depositions showed a negative correlation with mean soil moisture, but this was only significant for the buried juvenile remains (−0.81, *p* = 0.014). The buried remains also showed significant correlations with both mean soil temperature (0.72, *p* = 0.043) and ADD (0.87, *p* = 0.005). All the simple linear regression correlations are presented in Table 6.

## 4. Discussion

The morphology of bone bioerosion observed here is largely restricted to enlarged osteocyte lacunae, with extensive damage noted as amalgamations of the enlarged lacunae. This is similar to the morphology observed by White and Booth [22] in a *Sus scrofa* sample from Riseholme, United Kingdom. While this deviates from Hackett’s [44] MFD, it is consistent with the microstructural damage observed in studies with diffuse-bone demineralization resulting from corrosive environments [45,61,62]. Corrosive environments resulting in acidic bone dissolution can be promoted by anoxic-reducing environments, which is possible at the Lake Wheeler site [2,61]. Enlarged osteocyte lacunae can also appear when there is exogenous staining (i.e., discoloration) of bone microstructures, but here enlarged osteocyte lacunae are observed within as well as outside of regions of staining and display consistent coloration distinct from regions of staining [22,24]. This suggests that the observations are not artifacts of bone staining.

The results presented in this study indicate that microstructural damage can be observed as early as three months after deposition, which is consistent with that seen by Bell and colleagues [14] in adult bone in a forensic context. This suggests that factors affecting bone quality for subsequent testing (e.g., DNA or isotopic analyses) could be present early in the postmortem period in juvenile or fetal bone as in adult bone. Bone bioerosion, observed as tunneling or the amalgamation of enlarged osteocyte lacunae, did show some variation across depositional modes, which suggests that deposition can influence the extent or rate of microbial attack. For example, more extensive bone bioerosion was observed in the buried juvenile samples relative to the other depositions, but the morphology of the microstructural damage was similar across all samples. Further, the morphology of the microstructural damage observed here was similar to that observed by White and Booth [22] in their juvenile and neonatal study sample from Riseholme, United Kingdom. This suggests that while the observed morphology of bone bioerosion is similar across samples and environments [2,22,38,52], the factors that contribute to microbial invasion are more variable. Thus, a limiting factor is likely an intrinsic property related to how bone is accessed and degraded by microbial enzymes and not a shared extrinsic variable. Although one possible extrinsic variable that does influence the extent of microbial attack is deposition, Overall, the bag deposition showed the best preservation, while the buried samples showed the greatest extent of microbial attack.

In samples showing minimal microstructural damage (TI = 1) and minor microstructural damage with amalgamations of enlarged osteocyte lacunae (TI = 2), the damage was either only present or more extensive in the subendosteal region. In samples that showed major microstructural damage from amalgamations (TI = 3), the damage was observed either in both the subendosteal and subperiosteal regions or as a coalescence between these two regions with little to no involvement of the endosteal and periosteal surfaces. This is consistent with samples from archaeological and modern remains where non-Wedl MFD is concentrated in areas with apparent enlarged osteocyte lacunae [14,24]. The distribution of microstructural damage observed here is also consistent with archaeological samples that have shown little bone bioerosion [24,61]. Although studies of pig bones recovered from archaeological contexts have not noted other forms of bioerosion outside of non-Wedl MFD [42,63], it is possible that the morphology observed here is an early form of MFD, especially considering that many of the amalgamations show similar morphology to lamellate and linear longitudinal MFD.

The distribution of microstructural damage noted above also has implications for the source of microbial activity. Considering that both early and more extensive microstructural damage appear initially along the subendosteal region, it suggests that endogenous microbes were initially the primary source of attack. Further, the greatest destruction was seen in buried samples, with the coalescence of damage between the subendosteal and subperiosteal regions, while other depositions showed most damage in the subendosteal region even when extensive damage is present. This suggests two possibilities. The first possibility is that endogenous bacteria having access early in the postmortem period are primarily responsible for early observations of bioerosion, but when skeletonization occurs, bacterial activity becomes limited.

The second possibility is that early bioerosion is initiated by endogenous microbes, but soil exposure in a burial environment also introduces exogenous microbes in the later postmortem period as well as increased exposure to endogenous microbes as decomposition generally proceeds more slowly in buried remains. Both possibilities are also supported by the notion that chemical alteration of the mineral component is necessary for microbial attack to occur, and chemical processes are often the result of groundwater and surrounding soil chemical exchange [25,41,52,62]. Therefore, the more extensive degradation seen in the buried juvenile samples could have been facilitated by chemical processes that encouraged greater endogenous bacterial attack or by both endogenous and exogenous microbes as additional chemical exchange occurred with the surrounding soil. One caveat presented by this study is that bone bioerosion was also observed in the stillborn fetal remains as well, which deviates from previous studies [22]. While it has been stated that stillborn remains likely lack internal bacterial colonies, more recent research suggests this may not be as straightforward as previously thought [64]. Thus, the absence of bone bioerosion in neonatal or fetal remains may not be a conclusive indicator that bone bioerosion is of endogenous origin.

Finally, the positive linear relationship observed between ADD and the TI scores in this study suggests that there is a strong relationship between time and the extent of microstructural damage. Based on the destructive degradation model, it is likely the samples here would have exhibited complete microstructural degradation within a few years. This has implications for the survival of biomolecules used in forensic analyses, particularly for juvenile or fetal remains recovered from burial environments. Further, considering that most archaeological samples show good histological preservation [23,24,38,61,65], this suggests that the persistence of osseous structures in the archaeological and fossil records is likely determined early in the postmortem period.

## 5. Conclusions

The results of this study suggest that microstructural degradation may be a reliable indicator of the postmortem interval in forensic contexts where deposition and other extrinsic factors can be more reliably assessed. This is particularly important for the forensic recovery of fetal and juvenile remains, which will require molecular analyses. The morphology and distribution of microstructural damage across depositions suggest two scenarios of microbial attack: (1) The early appearance of damage in the subendosteal regions, early in the postmortem period, in all depositions suggests that the microbes responsible are of endogenous origin; (2) There is a combination of endogenous and exogenous invasion, with the exogenous attack occurring when remains can interact with the surrounding soil and groundwater, thereby increasing access to the bone microstructure by external sources as well.

## Figures and Tables

**Table 1:** Details of the environmental variables for the *Sus scrofa domesticus* sample used in this study. The end date for all study samples was 15 June 2015. ADD = Accumulated degree days (in °C). Mean soil temperature is reported in Fahrenheit. Mean soil moisture is reported in m^3^m^−3^.

Sample	Start Date	ADD	ADD (Buried)	Mean Soil Temperature	Mean Soil Moisture
Summer 2013	22 May 2013	11,966.61	12,558.61	61.24	0.26
Fall 2013	13 September 2013	9163.86	9630.06	59.64	0.27
Winter 2013	5 December 2013	7965.17	8252.11	58.06	0.28
Spring 2014	30 March 2014	7246.89	7494.78	61.17	0.31
Summer 2014	6 June 2014	5969.67	6171.67	60.48	0.30
Fall 2014	27 September 2014	3184.39	3378.39	55.96	0.33
Winter 2014	20 December 2014	2153.72	2221.56	52.76	0.33
Spring 2015	13 March 2015	1753.69	1748.94	59.35	0.33

**Table 2:** The definitions of the ordinal tunneling index (TI) used in this study and adapted from White and Booth [22]. Regions as defined here refer to the following: periosteal surface, subperiosteal region, subendosteal region, and endosteal surface.

Score	Category	Definition
0	No damage	Microstructure appears intact, with no enlarged osteocyte lacunae apparent.
1	Minor damage	Microstructure shows enlarged osteocyte lacunae not coincident with exogenous staining but no amalgamations.
2	Minor damage with amalgamation	Microstructure shows amalgamations of enlarged osteocyte lacunae not coincident with exogenous staining, but no coalescence or damage to more than one region of the microstructure.
3	Major damage	Microstructure shows amalgamations of enlarged osteocyte lacunae not coincident with exogenous staining, and coalescence of amalgamations across more than one region of the microstructure.

**Table 3:** Probabilities determined by the destructive degradation model for each deposition and the Bayesian informative criterion (BIC) of each log-logistic model. It says that there is a 63% probability that the tunneling index (TI) of bone would be a score of 2 for an accumulated degree day (ADD) of 6860.15. Time equivalence is based on the days of the study and the ADD for each deposition.

Deposition	Probability	TI	ADD	Time Equivalent	BIC
Bag fetal	0.63	2	6860.15	355 days	18.050
Blanket fetal	0.31	2	6860.15	355 days	21.539
Buried juvenile	0.25	2.5	7153.78	394 days	7.483
Surface juvenile	0.50	2	6860.15	355 days	16.740

**Table 4:** The model from the destructive degradation profile of each deposition with the log-likelihood for each model. TI = tunneling index. ADD = accumulated degree days.

Deposition	Model	Standard Deviation	Log-Likelihood
Bag fetal	TI = 0.222 + (5.407 × 10^−5^) × ADD	0.280	6.106
Blanket fetal	TI = 0.536 + (4.164 × 10^−5^) × ADD	0.199	7.650
Buried juvenile	TI = 0.621 + (5.032 × 10^−5^) × ADD	0.069	0.622
Surface juvenile	TI = 0.530 + (2.420 × 10^−5^) × ADD	0.176	5.251

**Table 5:** Model estimates from the partial least squares regression analysis testing the relative effects of ADD, mean soil temperature, and mean soil moisture on the tunneling index (TI).

Deposition	Model Parameter	Log Worth	*p*-Value (α = 0.05)
Bag fetal (R^2^ = 0.83)	Mean Soil Temperature	1.251	0.06
	Mean Soil Moisture	0.538	0.29
	ADD	0.432	0.37
Blanket fetal (R^2^ = 0.54)	Mean Soil Temperature	0.544	0.29
	Mean Soil Moisture	0.312	0.44
	ADD	0.356	0.49
Buried juvenile (R^2^ = 0.81)	Mean Soil Temperature	0.425	0.38
	Mean Soil Moisture	0.127	0.75
	ADD	0.495	0.32
Surface juvenile (R^2^ = 0.51)	Mean Soil Temperature	0.708	0.20
	Mean Soil Moisture	0.069	0.85
	ADD	0.359	0.44

**Table 6:** Correlation statistics and *p*-value for the relationship between the average soil temperature and the tunneling index scores by deposition. Bold values represent significant correlations (α = 0.05).

Variable	Bag Fetal	Blanket Fetal	Buried Juvenile	Surface Juvenile
Mean Soil Temperature	**0.85 (0.016)**	0.67 (0.067)	**0.72 (0.043)**	−0.12 (0.782)
Mean Soil Moisture	−0.42 (0.350)	−0.45 (0.267)	**−0.81 (0.014)**	−0.43 (0.290)
ADD	0.49 (0.260)	0.54 (0.168)	**0.87 (0.005)**	0.50 (0.253)

## Data Availability

Data is available upon request to ahross@ncsu.edu. Suggested Data Availability Statements are available in the section “MDPI Research Data Policies” at https://www.mdpi.com/ethics.
